# Immune reconstitution and infections with ATG versus post-transplant cyclophosphamide as GvHD prophylaxis after nonmyeloablative matched unrelated donor hematopoietic stem cell transplantation

**DOI:** 10.3389/fimmu.2026.1739320

**Published:** 2026-08-06

**Authors:** Mesire Aydin, David C. de Leeuw, Man Wai Tang, Caroline E. Rutten, Otto J. Visser, Cinthy van Roessel, Jeroen J.W. Janssen, Bart J. Biemond, Arjan A. van de Loosdrecht, Mette D. Hazenberg, Erfan Nur

**Affiliations:** 1 Department of Hematology, Amsterdam UMC, University of Amsterdam, Amsterdam, Netherlands; 2 Department of Hematology, Amsterdam UMC, Free University, Amsterdam, Netherlands; 3 Cancer Center Amsterdam, Amsterdam University Medical Center, Amsterdam, Netherlands; 4 Department of Hematology, Isala Hospital, Zwolle, Netherlands; 5 Department of Hematology, Radboud University Medical Center, Nijmegen, Netherlands; 6 Department of Hematopoiesis, Sanquin Research and Landsteiner Laboratory, Amsterdam, Netherlands; 7 Department of Molecular Hematology, Sanquin Research and Landsteiner Laboratory, Sanquin Research, Amsterdam, Netherlands

**Keywords:** admission, allogeneic transplantation, ATG, infection, PTCY, reactivation

## Abstract

**Introduction:**

Anti-thymocyte globulin (ATG) and post-transplantation cyclophosphamide (PTCy) are both used as *in-vivo* lymphodepleting treatment in allogeneic hematopoietic stem cell transplantation (HSCT), particularly in the matched unrelated donor (MUD) setting.

**Methods:**

In this retrospective study, we compared the incidences of infections and viral reactivations and evaluated immune cell reconstitution in the first-year post-transplant between ATG- and PTCy-based conditioning regimens in nonmyeloablative MUD transplantations. Infection incidence was evaluated for the periods 0-100 vs. 101-365 days post-transplant.

**Results:**

A total of 185 patients (ATG=95, PTCy=90), transplanted between 2014 and 2021, were included. In the first 100 days after transplantation, the cumulative incidence of bacterial infections was higher in the PTCy group (ATG 17% vs. PTCy 32%, p=0.01) while CMV reactivations were more frequent in the ATG group (36% vs. 16%, p<0.001). Beyond 100-days post-transplant, incidence of bacterial infections was higher in the ATG group (18% vs. 1%, p<0.001). Fungal infection incidence was similar in both groups/periods. The 1-year densities of viral infections (3.54 vs. 1.89, p<0.01) and CMV reactivations (2.21 vs. 0.68, p<0.01) were higher in the ATG compared to the PTCy group. After 1-year, circulating CD4+ T-cell counts were significantly lower in the ATG than in the PTCy group (p<0.001).

**Discussion:**

In conclusion, this study shows that overall PTCy is associated with better immune reconstitution and reduced rate of viral infections/CMV reactivations compared to ATG-based conditioning. Together with our previous findings of a lower risk of acute GvHD following PTCy compared to ATG-based conditioning, these data may favor PTCy over ATG as GvHD prophylaxis.

## Introduction

Allogeneic hematopoietic stem cell transplantation (HSCT) is a potentially curative treatment for patients with hematological disorders. Nonmyeloablative conditioning (NMC) regimens are increasingly used in transplantations due to their lower toxicity profile as compared to myeloablative regimens (1). This has resulted in fewer early infections and reduced transplantation-related toxicity (2, 3). Over the years, significant improvements have been made in graft-versus-host disease (GvHD) prophylaxis and supportive care to improve HSCT outcomes (4, 5). Both anti-thymocyte globulin (ATG) and post-transplant cyclophosphamide (PTCy) are commonly used as lymphocyte depleting strategies in allogeneic HSCT to decrease the risk of GvHD, especially when using a matched unrelated donor (MUD) (6–9). While lymphodepletion by ATG or PTCy may be effective in reducing GvHD incidence, it also increases the susceptibility to infections following transplantation (10, 11). Post-transplantation infections contribute significantly to the non-relapse mortality (NRM) after allogeneic HSCT (12, 13). Various studies have compared transplantation outcomes between ATG- and PTCy-containing conditioning regimens for MUD transplantations and although it was demonstrated that PTCy and ATG are associated with a significantly restricted T cell receptor repertoire until 2 years after HSCT (14, 15), very little is known about the differences in immune reconstitution and infectious complications between these two strategies (16–18). We recently showed that ATG was associated with an increased risk of acute GvHD compared to PTCy in nonmyeloablative MUD transplantations (18). In this retrospective study, we evaluated clinically relevant infections necessitating medical intervention and/or hospitalization, and reconstitution of T, B and NK cells in adult patients with hematological malignancies who received a 10/10 MUD transplantation with ATG- or PTCy-based nonmyeloablative conditioning.

## Methods

### Study design and population

This is a retrospective analysis of transplantation outcomes in consecutive adult patients receiving a nonmyeloablative MUD transplantation between January 2014 and December 2021 at two academic hospitals in Amsterdam (Academic Medical Center (location A) and VU Medical Center (location B)) that have recently merged into one center (Amsterdam UMC). Both centers used comparable supportive care protocols, including antimicrobial/antiviral/antifungal prophylaxis. Cytomegalovirus (CMV) was monitored weekly in patients who were recipient and/or donor CMV seropositive until at least day +100 post-transplant. The CMV monitoring frequency decreased to 2-weekly and later to approximately 4–6 weekly between day +100 and day +365 unless the patient had to continue immunosuppressive therapy due to GvHD. Patients were treated with valganciclovir 900 mg orally or ganciclovir 5 mg/kg iv. twice daily, when CMV reactivation, defined as ≥1000 CMV copies/mL using serum quantitative polymerase chain reaction (qPCR). Patients not tolerating ganciclovir or with ganciclovir resistant CMV reactivation/infection were treated with intravenous foscarnet. None of the patients received letermovir as prophylaxis. As per institutional protocol, patients treated with ATG were discharged on day +1 or +2 post-transplantation, while patients treated with PTCy remained hospitalized until their absolute neutrophil count (ANC) recovered to above 0.5 x 10^9^/L. Numbers of circulating neutrophils, thrombocytes and T, B and NK cells were measured routinely as part of standard clinical operating procedures and were retrospectively extracted from the medical records.

### GvHD prophylaxis

In the ATG group, patients received cyclosporine starting on day −3 (target trough level 200–300 ng/mL) in combination with mycophenolate mofetil (15 mg/kg every 8 hours) from day 0 to day +84. In the absence of GvHD, cyclosporine was tapered from day +100 onwards. In the PTCy group, patients received intravenous cyclophosphamide (50 mg/kg) on days +3 and +4, followed by cyclosporine (target trough level 200–300 ng/mL) from day +5 to day +70.

### Study endpoints and definitions

The neutrophil and platelet engraftment were defined as ANC of >0.5 x 10^9^/L and platelets >20 x 10^9^/L after the nadir without platelet transfusion. Death before day 30 post-transplantation was considered a competing event. Infections were retrospectively identified from the day of stem cell infusion (day 0) up to 1-year post-transplantation and were categorized as bacterial, fungal, and viral infection (CMV reactivation was registered separately), as described previously (19). Bacterial and fungal infections were defined based on clinical presentation supported by microbiological findings, and viral infections were defined based on PCR detection with or without clinical symptoms, depending on the pathogen. Only clinically relevant infections requiring medical intervention and/or hospitalization were included. CMV reactivation was defined as a CMV viral load (≥1000 copies/ml) without signs of CMV infection, with the day of sampling being considered as the onset of the reactivation. Epstein–Barr virus (EBV) reactivation was defined as detection of EBV DNA in peripheral blood by quantitative PCR, with the date of first detection considered as the onset of reactivation. Pre-emptive therapy was initiated at an EBV DNA load ≥10³ IU/mL. Pneumonia was accepted as an infection in the presence of both clinical and radiological findings of pneumonia with or without microbiologic confirmation. Fever of undetermined origin (whether during neutropenia or not) was not considered to be an infectious complication. The cumulative incidences of bacterial, fungal, and viral infections as well as CMV reactivation were calculated based on the time-to-first infection, while death without infections was considered a competing risk in the first year. The analysis of infection density - which accounts for multiple infectious events in a patient - was calculated by determining infection frequencies over 1000 patient days at risk. Cumulative incidence and infection density over 2 periods (days 0 to +100 and day +101 to +365) and the whole period (0 to + 365) were compared between the treatment groups. The incidence of CMV disease, EBV reactivation, and EBV-related post-transplantation lymphoproliferative disorder were reported separately. All available data of immune reconstitution (T-cells, NK cells and B-cells) as measured by flow cytometry post-transplantation were analyzed and compared between the groups. Transplantation-related readmissions in the first year were divided into two periods (day 0 to +100 and day +101 to +365) and were summarized per cause of readmission. Hospitalization-free days were defined as days alive, without relapse and out of the hospital in the first-year post-transplantation. The total admission days between the conditioning regimen and engraftment and readmission days post-transplantation were subtracted from 365 days to calculate hospitalization-free days. Patients who died before day +365 were followed from day 0 until the time of death.

### Statistical analysis

Continuous variables were assessed using the non-parametric test (Mann–Whitney U test) and categorical variables were analyzed using the Chi-square test (or Fisher’s exact). The incidences of infections and engraftment between the groups were compared using Gray’s method for competing risk analysis. Covariates associated with infections were explored using the Cox proportional hazards regression model, and the models were tested using Schoenfeld residuals test to explore violations of this assumption. The following covariates were assessed in both univariate and multivariate regression models: ATG versus PTCy, age (≤40 vs. >40 years), diagnosis, occurrence of GvHD and hospital readmissions. Results are expressed as a hazard ratio (HR) with a 95% confidence interval (95% CI). To compare infection densities between time periods and groups, the rate ratio was calculated using the mid-P method. Hospitalization-free days for both groups were calculated using the Wilcoxon signed-rank test. SPSS Statistics 27.0 (IBM Corporation, New York, USA) was used for the baseline characteristics and R 3.5.1 (R Core Team [2020]) for the survival curves (20, 21). The data that support the findings of this study are available from the corresponding author upon reasonable request.

## Results

### Baseline characteristics and transplantation outcomes

Patient and transplantation characteristics are summarized in **Table 1**. Between January 2014 and December 2021, 185 adult patients diagnosed with hematological malignancies were transplanted with 10/10 MUD using nonmyeloablative conditioning regimens with either ATG (n = 95) or PTCy (n = 90) as *in vivo* lymphodepleting method in two transplantation centers in Amsterdam. All patients receiving ATG-based conditioning and 4 patients receiving PTCy-based conditioning were transplanted at location A, the remaining 86 patients receiving PTCy-based transplantations were treated at location B. The ATG-containing conditioning regimen consisted of fludarabine (total dose 90 mg/m^2^), ATG (total dose 8 mg/kg) and 200 cGy total body irradiation (TBI). The PTCy-based conditioning regimen consisted of cyclophosphamide (total dose 29 mg/kg), fludarabine (total dose 150 mg/m^2^), 200 cGy TBI and PTCy (50 mg/kg on days +3 and +4) in the majority of patients (96%), while 4 patients (4%) received fludarabine (total dose 150 mg/m^2^) and TBI 200 cGy with PTCy. Transplantation outcomes have been reported previously (18). In brief, the 1-year cumulative incidence of grade II–IV acute GvHD was significantly higher in the ATG group compared with the PTCy group (48% vs. 21%, respectively; p < 0.001). Overall survival was higher in the PTCy group, although this did not reach statistical significance (3-year OS: 68% vs. 55% in the ATG group; p = 0.12).

**Table 1:** Baseline characteristics.

Characteristic	ATG (n = 95)	PTCy (n = 90)	P-value
Age, median (IQR)	60 (52 – 66)	57 (48 – 66)	0.43
Disease risk‡, n (%)			0.29
Non-adverse risk	38 (40)	43 (48)	
Adverse risk	57 (60)	47 (52)	
Diagnosis, n (%)			0.58
AML/MDS	62 (65)	54 (60)	
ALL	6 (6)	11 (12)	
CLL/HL/NHL	24 (25)	22 (24)	
CML/CMML	3 (3)	3 (3)	
Disease response at HSCT, n (%)			0.78
CR1	54 (57)	58 (64)	
≥CR2	25 (26)	20 (22)	
PR	7 (7)	4 (5)	
SD/Upfront	9 (10)	8 (9)	
WHO performance score, n (%)			0.11
0-1	93 (98)	83 (92)	
2	2 (2)	7 (8)	
HCT-CI, n (%)			0.11
0-1	41 (43)	50 (56)	
≥2	54 (57)	40 (44)	
CD34+ cell dose, median (IQR)	7.0 (5.7 – 8.9)	7.1 (5.6 – 9.5)	0.53
CD3+ cell dose, median (IQR)	259 (201 – 335)	258 (188 – 329)	0.65
CMV serostatus R/D			0.22
+/+	43 (45)	35 (39)	
+/-	8 (9)	17 (19)	
-/+	7 (7)	6 (7)	
-/-	37 (39)	32 (35)	

ATG, anti-thymocyte globulin; PTCy, post-transplantation cyclophosphamide; AML, acute myeloid leukemia; ALL, acute lymphoblastic leukemia; MDS, myelodysplastic syndrome; CLL, Chronic lymphoblastic leukemia: CML, Chronic myeloid leukemia; CMML, Chronic myelomonocytic leukemia; HL, Hodgkin lymphoma; NHL, non-Hodgkin lymphoma; CR; complete remission; PR, partial remission; SD, stable disease; WHO, world health organization; HCT-CI, hematopoietic stem cell transplantation comorbidity index, R/D; recipient/donor, CMV; cytomegalovirus, IQR; interquartile range.

‡ Disease risk is divided in non-adverse (low/intermediate), and adverse (high/very high) risk based on diagnoses, disease status and cytogenetic/molecular abnormalities.

### Engraftment

Graft failure was not observed in any of the participants during the first year post-transplantation. The median time to neutrophil engraftment was 20 days [interquartile range (IQR) 15 – 24] in the ATG group compared to 22 days (IQR 19 – 26) in the PTCy group (p = 0.036). The cumulative incidence of neutropenia (ANC <0.5 x 10^9^/L) prior to engraftment was 60% in the ATG group compared to 96% in the PTCy group (p<0.001). The median time to platelet engraftment was 19 days (IQR 15 – 22) in the ATG group compared to 22 days (IQR 16 – 33) in the PTCy group (p = 0.07). The cumulative incidence of thrombocytopenia (platelet counts <20 x 10^9^/L) was 32% in the ATG group versus 83% in the PTCy group (p<0.001). Death before engraftment occurred in three patients (3.3%) due to infections (n = 2) and cardiogenic shock (n = 1) in the PTCy group and in one patient (1%) due to infectious complications in the ATG group.

### Infections

**Figure 1:**
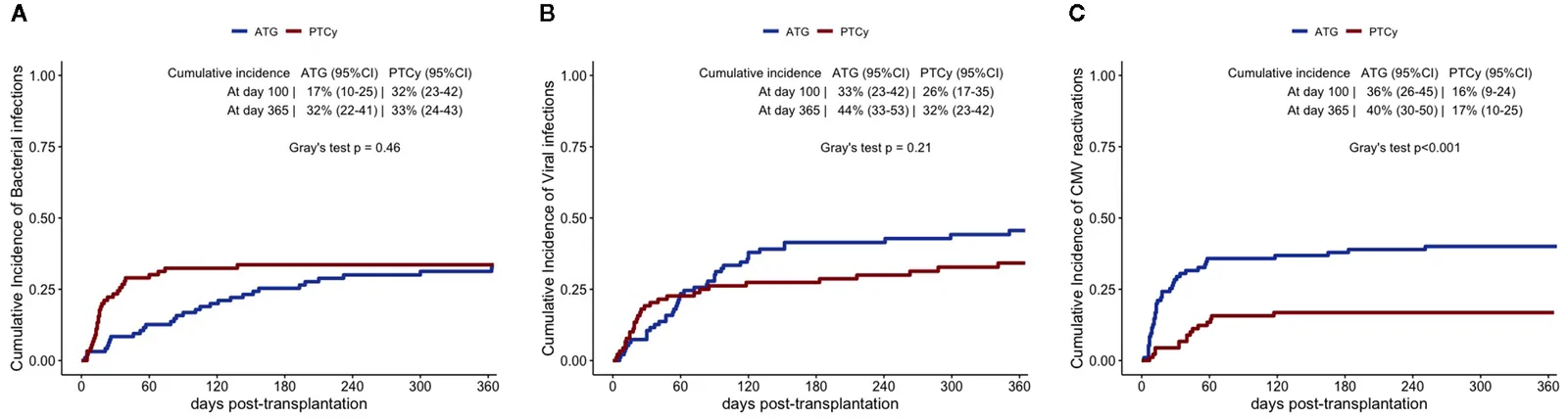
Cumulative incidence of bacterial infections **(A)**, viral infections **(B)** and CMV reactivations **(C)** until day +365.

**Figure 2:**
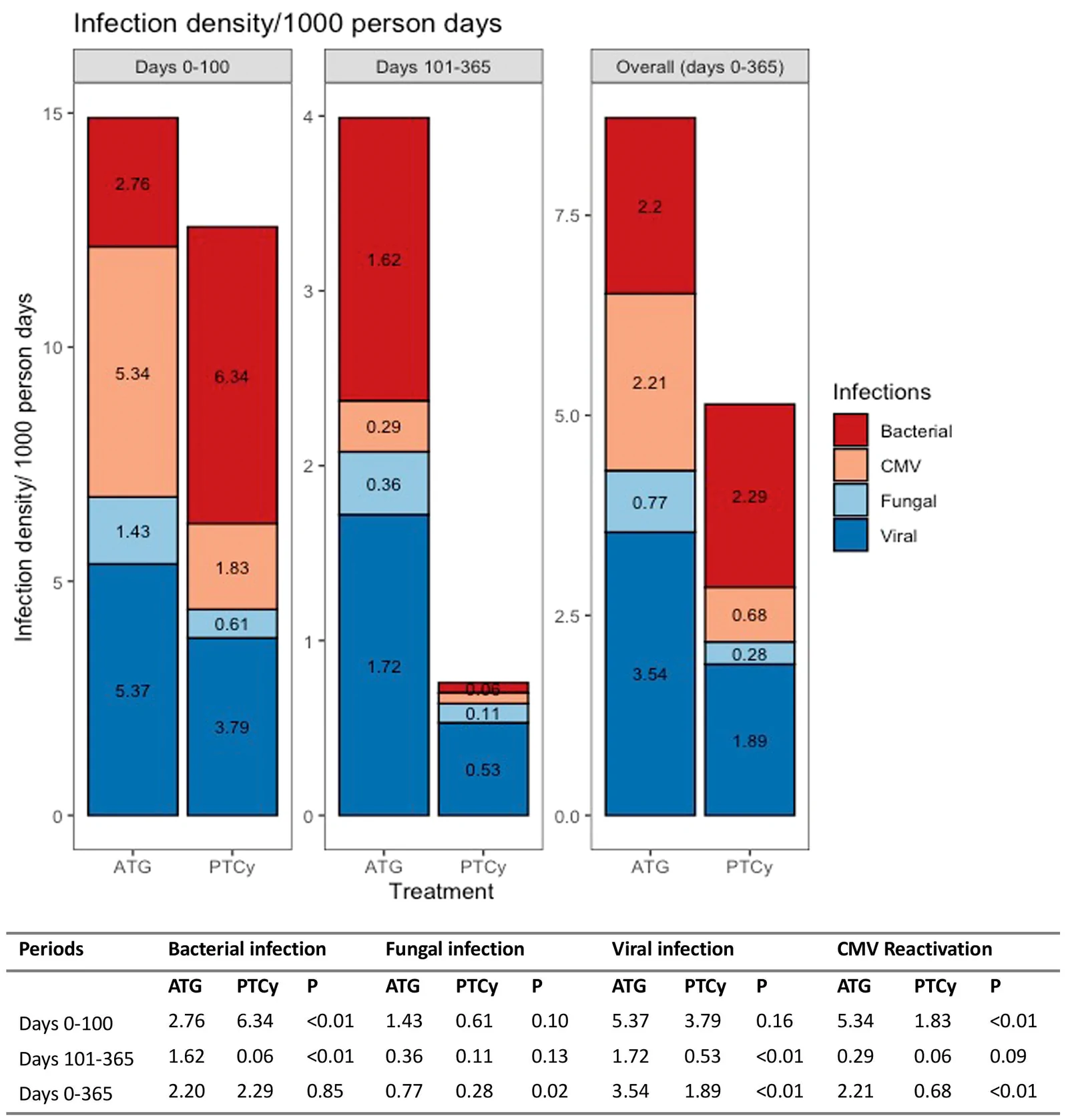
Infection density per 1000 person days per period under study.

The cumulative incidence of infections is shown in **Table 2**. Patients in the ATG group had a lower incidence of bacterial infections by day +100 compared to those in the PTCy group [17% (95%-confidence interval (CI) 10 – 25) vs. 32% (95%-CI 23 – 42), respectively, p = 0.01]. In contrast, the incidence of bacterial infections after day +100 was higher in the ATG group than in the PTCy group [18% (95%-CI 11 – 27) vs. 1% (95%-CI 1 – 6), p <0.001]. Both acute GvHD (HR 2.30, p = 0.003) and readmissions (HR 2.20, p = 0.005) were significantly associated with bacterial infections (**Table 3**). The cumulative incidence of fungal infections was comparable between both groups and periods. In multivariate analysis, only readmissions were significantly associated with fungal infections (HR 2.73, p = 0.043). Incidence of viral infections by day +100 was similar in both treatment groups (p = 0.44), while patients in the ATG group developed more viral infections after day +100 [22% (95%-CI 14 – 31) vs. 10% (95%-CI 5 - 17), p = 0.022]. In multivariate analysis, viral infections were more frequent in patients with AML/MDS (HR 1.79, p = 0.02), acute GvHD (HR 1.67, p = 0.042) and readmissions (HR 2.21, p = 0.003). The cumulative incidence of CMV reactivation by day +100 was higher in the ATG group than in the PTCy group [36% (95%-CI 26 – 45) vs. 16% (95%-CI 9 – 24), p<0.001; **Figure 1**]. The cumulative incidence of CMV active disease was 8% (n = 8) in the ATG group and 0% in the PTCy group (p = 0.005). In multivariate analyses, PTCy was associated with a significantly reduced risk of CMV reactivation (HR 0.44, p = 0.012). The cumulative incidence of EBV reactivation was 5 (6%) in the ATG group, and 0% in the PTCy group (p = 0.01). Two of the five patients with EBV reactivation in the ATG group developed EBV-related post-transplantation lymphoproliferative disorder. Densities of infections and CMV reactivations are shown in **Figure 2**. Infection density analyses showed higher bacterial infection density (per 1000 person days) by day +100 in the PTCy group than in the ATG group (2.76 in ATG vs. 6.34 in PTCy group respectively, p<0.01) while after day +100 this was higher in the ATG group (1.62 in ATG vs. 0.06 in PTCy group respectively, p<0.01). The overall density (day 0 to +365 post-transplant) for bacterial infections was balanced between the groups. The density of fungal infections was comparable by day +100 (1.43 vs. 0.61, p = 0.10) and after day + 100 (0.36 vs. 0.11, p = 0.13), while the overall density was significantly higher in the ATG group (0.77 vs. 0.28, p = 0.02). Density of viral infections was significantly higher in the ATG group than in the PTCy group in the day +101 to +365 period (1.72 vs. 0.53, p<0.01) and in the overall density analysis in the first year post-transplantation (3.54 vs. 1.89, p<0.01). Density of CMV reactivations was higher in the ATG group from day 0 to +100 (5.34 vs. 1.83, p<0.01) and in the overall density analysis (2.21 vs. 0.68, p<0.01), while it was relatively low in both groups after day+100 (0.29 vs. 0.06, p = 0.09). Infection-related mortality was observed in 13% (n = 13) of patients in the ATG group and 8% (n = 7) of patients in the PTCy group.

**Table 2:** Engraftment and cumulative incidence of infections.

Outcome	ATG (n = 95)	PTCy (n = 90)	p-value
Engraftment post-transplantation			
Cumulative incidence of neutropenia (ANC <0.5 x 10^9^/L), (95%-CI)	60% (49 – 69)	96% (88 – 98)	*<0.001*
Median ANC engraftment days (IQR)	20 (15 – 24)	22 (19 – 26)	0.036
Cumulative incidence of thrombocytopenia (platelets <20 x 109/L), (95%-CI)	32% (22 – 41)	83% (74 – 90)	<0.001
Median platelet engraftment days (IQR)	19 (15 – 22)	22 (16 – 33)	0.07
Cumulative incidence of infections %, (95%-CI) %, (95%-CI)			
Bacterial infections			
≤100 days	17% (10 – 25)	32% (23 – 42)	0.01
>100 days	18% (11 – 27)	1% (1 – 6)	<0.001
Overall (0–365 days)	32% (22 – 41)	33% (24 – 43)	0.46
Fungal infections			
≤100 days	12% (6 – 19)	6% (2 – 12)	0.14
>100 days	5% (2 – 11)	2% (1 – 7)	0.29
Overall (0–365 days)	17% (10 – 25)	8% (3 – 16)	0.06
Viral infections			
≤100 days	33% (23 – 42)	26% (17 – 35)	0.44
>100 days	22% (14 – 31)	10% (5 – 17)	0.022
Overall (0–365 days)	44% (33 – 53)	32% (23 – 42)	0.21
CMV reactivations			
≤100 days	36% (26 – 45)	16% (9 – 24)	0.008 0.19
>100 days	4% (1 – 10)	1% (0 – 5)	
Overall (0–365 days)	40% (30 – 50)	17% (10 – 25)	<0.001
CMV active disease*	8% (4 - 15)	–	0.005

ATG, anti-thymocyte globulin; PTCy, post-transplantation cyclophosphamide; 95%-CI, confidence interval; ANC, absolute neutrophil count; IQR, interquartile range; CMV, cytomegalovirus.

*CMV active disease includes CMV pneumonitis and colitis.

**Table 3:** Cox regression analysis for infections.

Covariate	Bacterial infections		Fungal infections		Viral infections		CMV reactivations	
	HR (95%-CI)	*P*	HR (95%-CI)	*P*	HR (95%-CI)	*P*	HR (95%-CI)	*P*
ATG vs. PTCy	1.20 (0.75 – 2.10)	0.46	0.57 (0.22 – 1.47)	0.25	0.92 (0.56 – 1.54)	0.76	0.44 (0.23 – 0.83)	*0.012*
Age (≤40, >40)	2.5 (0.79 – 8.10)	0.12	0.68 (0.20 – 2.34)	0.55	0.98 (0.46 – 2.05)	0.95	1.73 (0.62 – 4.81)	0.30
Diagnosis (AML/MDS vs. Other)	1.3 (0.79 – 2.20)	0.29	1.88 (0.80 – 4.40)	0.15	1.79 (1.11 – 2.89)	*0.02*	0.85 (0.48 – 1.51)	0.58
Acute GvHD*	2.3 (1.32 – 3.80)	*0.003*	1.24 (0.53 – 2.89)	0.62	1.67 (1.02 – 2.73)	*0.042*	1.42 (0.78 – 2.59)	0.25
Readmissions*	2.20 (1.27 – 3.80)	*0.005*	2.73 (1.03 – 7.22)	*0.043*	2.21 (1.32 – 3.69)	*0.003*	1.25 (0.67 – 2.32)	0.49

ATG, anti-thymocyte globulin; PTCy, post-transplantation cyclophosphamide; AML/MDS, acute myeloid leukemia/myelodysplastic syndrome; GvHD, graft-versus-host disease; CMV, cytomegalovirus; HR, hazard ratio; CI, confidence interval; P, p-value.

*Both acute GvHD and readmissions are calculated as time-dependent covariates.

### Immune reconstitution

**Figure 3:**
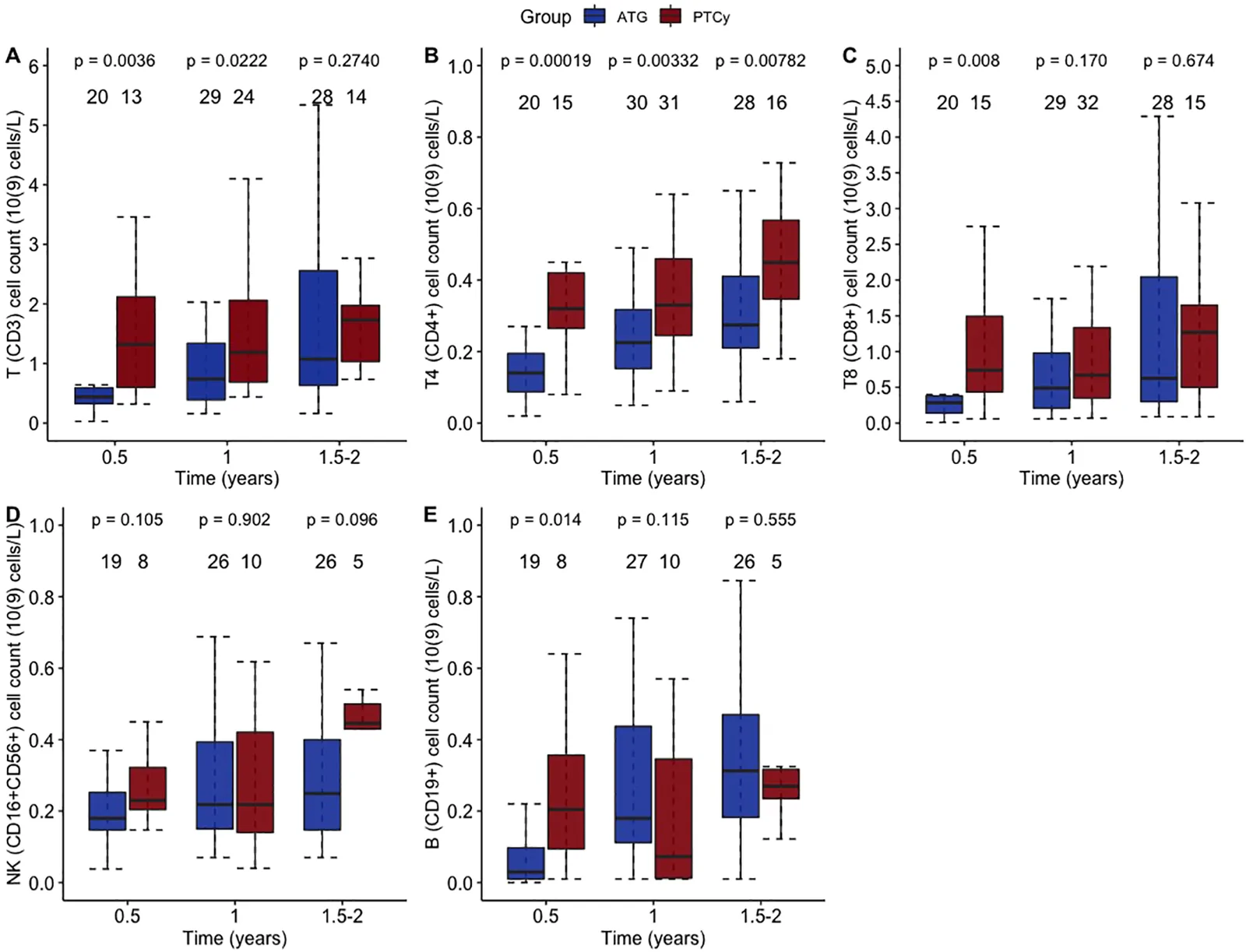
Lymphocyte reconstitution after allogeneic HCT. Circulating numbers of **(A)** CD3+ T cells, **(B)** CD4+ T cells, **(C)** CD8+ T cells, **(D)** CD16+CD56+ NK cells and **(E)** CD19+ B cells at around 6 months [mean ± standard deviation (SD) time in days: ATG 128 ± 24, PTCy 131 ± 29, p = 0.33], 1 year (mean ± SD time in days: ATG 299 ± 72, PTCy 325 ± 72, p = 0.11), and 1.5–2 years (mean ± SD time in days: ATG 523 ± 72, PTCy 511 ± 63, p = 0.59) in a selection of patients.

Approximately 6 months post-transplantation, total counts of circulating CD3^+^ T cells, CD4^+^ and CD8^+^ T cells and B cells were significantly higher in the PTCy group compared to the ATG group, while NK cell counts were comparable between both groups (**Figure 3**). At 1-year post-transplantation CD3^+^ and CD4^+^ T cell counts were still lower in the ATG group compared to the PTCy group, while CD4^+^ T cell numbers remained lower in the ATG group beyond the first-year post-transplantation (**Figure 3B**).

### Hospitalization days

The median length of stay during hospitalization for transplantation was 10 days (IQR 10 – 12) in the ATG group and 28 days (IQR 24 – 33) in the PTCy group (p< 0.001). A total of 55 (58%) patients in the ATG group and 22 (24%) patients in the PTCy group were readmitted at least once during the first year post-transplantation (p<0.001), while 23 (24%) patients in the ATG group and 7 (8%) patients in the PTCy group were readmitted more than once (p<0.001). The three major causes of readmission were infection (n = 56), acute or chronic GvHD (n = 44) and intravenous treatment of CMV reactivation (n = 10), as shown in **Table 4**. Readmissions for infectious complications were similar in both groups and periods. Readmissions for GvHD during the first 100 days post-transplantation were more common in the ATG group than in the PTCy group (p = 0.002). The number of hospitalization-free days, correcting for differences in length of stay during hospitalization for the transplantation itself and mortality in the first year post-transplantation, was 324 days (IQR 120 – 352) in the ATG group and 331 days (IQR 181 – 337) in the PTCy group (p=0.20).

**Table 4:** Causes of readmissions post-transplantation <1-year.

		ATG (n = 95)				PTCy (n = 90)				
Causes of admission	Time	N of unique patients, (%)	N of admissions	Median admission days, IQR	Total days	N of unique patients, (%)	N of admissions	Median admission days, IQR	Total days	p-value*
Infection	<100	16 (17)	20	4 (3 - 9)	169	11 (12)	15	8 (4 - 18)	280	0.37
	>100	8 (8)	14	9 (4 – 20)	158	4 (4)	7	4 (4 – 6)	29	0.27
Treatment of GvHD	<100	20 (21)	25	21 (5 – 57)	1030	5 (6)	5	9 (4 - 11)	39	*0.002*
	>100	11 (12)	13	14 (5 – 28)	275	1 (1)	1	1	1	*0.004*
Intravenous treatment of CMV reactivation	<100	5 (5)	8	15 (6 – 15)	102	0	0	–	–	0.06
	>100	2 (2)	2	5 (2 – 8)	10	0	0	–	–	0.49
Other**	<100	19 (20)	21	4 (2 - 6)	104	8 (9)	8	6 (2 – 11)	86	0.32
	>100	4 (4)	4	3 (2 – 4)	11	2 (2)	2	2 (1 – 3)	4	0.68

ATG, anti-thymocyte globulin; PTCy, post-transplantation cyclophosphamide; HSCT, hematopoietic stem cell transplantation; GvHD, graft-versus-host-disease; CMV, cytomegalovirus; N, number; IQR, interquartile range.

*p-value: comparison of the number of unique patients in both groups

**Other: includes admissions caused by gastrointestinal complications or fever without identifiable infection.

## Discussion

The present study showed that the use of PTCy as GvHD prophylaxis in patients undergoing nonmyeloablative allogeneic hematopoietic stem cell transplantation with a full matched unrelated donor was associated with a significantly lower incidence of viral infections and CMV reactivations. While bacterial infections before engraftment were more common in the PTCy group resulting in a slightly higher early transplant related mortality, bacterial infections occurred in the ATG group more frequently after engraftment. To our knowledge, this is the first detailed comparison of immune reconstitution and related complications between ATG- and PTCy-based conditioning regimens. Together with our previous observations of higher incidences of acute GvHD (p<0.001) in the ATG group (18), these data favor PTCy over ATG as GvHD prophylaxis in the setting of non-myeloablative or reduced intensity conditioning.

Given the higher viral infection density after 100 days post-transplantation and the higher incidence of CMV reactivation in the ATG group, ATG seems to result in a delayed immune reconstitution in this group compared to the PTCy group (23, 24). In line with this observation, the number of circulating CD4^+^ T cells remained lower in the ATG group during the period under study. This finding is consistent with previous comparisons of ATG- and PTCy-based prophylaxis reporting faster CD4^+^ T-cell recovery after PTCy, although recovery of other immune subsets may differ between platforms (25, 26). Studies in MUD transplantations have also shown that T-cell reconstitution after ATG- and PTCy-based prophylaxis is heterogeneous and subset-specific. In our cohort, delayed CD4^+^ T-cell recovery in the ATG group may have contributed to the higher incidence of viral infections and CMV reactivations. However, direct comparison between studies remains limited by differences in ATG exposure, PTCy regimens, conditioning intensity, CMV prophylaxis and recent letermovir use (27, 28).

Several studies have indicated that immunosuppression as treatment of GvHD (primarily systemic steroids) increases the risk of CMV reactivation, while a bidirectional relationship has also been suggested where CMV reactivation induces inflammation, thereby increasing the risk of developing acute GvHD (23, 24, 29).

Although CMV reactivation was analyzed as a binary outcome in our study, previous studies have shown that CMV viral load kinetics may reflect the clinical impact of CMV, including the risk of CMV disease and survival outcomes after allogeneic transplantation (30, 31).

In addition, the observed association between readmission and fungal infections likely reflects a subgroup of patients with a more complicated post-transplant course, including GvHD, prior infections, and intensive immunosuppressive therapy, rather than a causal relationship.

A retrospective study comparing ATG and PTCy-based conditioning regimens found comparable incidences of CMV reactivation between both groups, while EBV reactivations were significantly more frequent in the ATG group (5% vs. 0%, p = 0.05) (22). The incidence of infections and the rate of readmissions were not reported in that study. A recent systematic review comparing ATG and PTCy for unrelated donor transplantations reported no differences in CMV reactivations between the groups (32). However, it did demonstrate that the risk of developing EBV-related post-transplant lymphoproliferative disease was higher in patients receiving ATG than in those treated with PTCy-based regimens. In the current study, EBV reactivations necessitating treatment (6%) and EBV-related post-transplant lymphoproliferative disease (2%) were only observed in the ATG group.

While the median time to neutrophil engraftment was significantly shorter in the ATG group, it was relatively long with a median duration of 20 days. This might be mainly explained by the less frequent monitoring in the ATG group, as patients in this group were discharged on day +1, while patients in the PTCy group remained in hospital until engraftment. To better capture the difference in duration of neutropenia between the groups, we also calculated the cumulative incidence of neutropenia (ANC <0.5 x 10^9^/L) prior to engraftment, which was significantly lower in the ATG group (60%) compared to the PTCy group (96%).

A recent study suggests that ATG dosing based on absolute lymphocyte count (ALC), alongside body weight, may reduce toxicity and improve outcomes of ATG-based strategies (33). In the ATG cohort in the current study, ALC was not used and therefore not routinely measured upon ATG administration, Whether ALC-based ATG dosing might result in more favorable outcomes than PTCy, remains to be elucidated in future studies.

Although both centers used harmonized supportive care protocols and treated patients from the same region, the inclusion of patients from two different centers may have introduced bias, including potential center-related batch effects and local differences in clinical decision-making.

Infections were identified based on databases and medical records, which made it challenging to assess the clinical severity of complications. Although we only included data from hospital visits, patients were closely monitored during the first year after transplantation. Furthermore, given that one of our inclusion criteria was a minimum follow-up period of one year, we focused solely on presenting results for this specific timeframe, while post-transplantation complications may have also occurred beyond one year. While EBV monitoring was performed periodically in the ATG group, it was not done routinely in the PTCy group. This might have resulted in missing temporary EBV reactivations in the PTCy group.

In conclusion, this study provides data suggesting that PTCy based conditioning is associated with earlier immune reconstitution, reduced rate of viral infections and CMV reactivations compared to ATG-based conditioning. Together with our previous findings of a lower risk of acute GvHD with PTCy than with ATG-based conditioning, these data again may favor PTCy over ATG as GvHD prophylaxis. Prospective studies that are sufficiently powered are needed to further dissect risks and advantages of the use of ATG and PTCy as GvHD prophylaxis.

## Data Availability

The original contributions presented in the study are included in the article/**Supplementary Material**. Further inquiries can be directed to the corresponding author.
