# Supplementary material for: Immune reconstitution and infections with ATG versus post-transplant cyclophosphamide as GvHD prophylaxis after nonmyeloablative matched unrelated donor hematopoietic stem cell transplantation
Source: Front Immunol. 2026 Aug 6;17:1739320. doi: 10.3389/fimmu.2026.1739320 (PMC13489835; doi:10.3389/fimmu.2026.1739320)
Supplement: Supplementary file 1 [file Table1.docx]

**Supplementary data**

Table S1. Infections divided in subgroups based on location and grade^1^

| Infections | ATG N = 95, N (%) | PTCy, N= 90, N (%) |
| --- | --- | --- |
| Bacterial, N  Bacteremia  Non-bloodstream infection*  Gastro-intestinal | *49*  20 (41)  21 (43)  8 (16) | *42*  22 (52)  15 (36)  5 (12) |
| Viral, N  Respiratory  Herpes  BK-virus  Gastro-intestinal | *67*  30 (44)  14 (21)  17 (25)  7 (10) | *34*  22 (65)  2 (6)  9 (26)  1 (3) |
| Fungal, N  Candida  Aspergillus | *18*  12 (67)  6 (33) | *7*  2 (29)  5 (71) |
| ** Urinary tract, lung and skin* | | |
| Bacterial, N  Grade 1  Grade 2  Grade 3 | *49*  14 (29)  25 (51)  10 (20) | *42*  7 (17)  29 (69)  5 (14) |
| Viral, N  Grade 1  Grade 2  Grade 3 | *68*  29 (43)  36 (53)  2 (3) | *34*  11 (32)  23 (68)  - |
| Fungal, N  Grade 1  Grade 2  Grade 3 | *18*  8 (44)  4 (22)  6 (33) | *7*  2 (29)  -  5 (71) |
